# Efficacy of Dupilumab in severe Atopic Dermatitis (AD) using the AD Control Tool (ADCT) Score in the population of Qatar

**DOI:** 10.5339/qmj.2023.sqac.11

**Published:** 2023-11-19

**Authors:** Febu Joy, Sara AlKhawaga, Maryam Iqneibi, Fahad Al Marri, Radi Al Chalabi, Joerg Buddenkotte, Martin Steinhoff

**Affiliations:** ^1^Department of Dermatology and Venereology, Hamad Medical Corporation, Doha, Qatar Email: MSteinhoff@hamad.qa; ^2^Dermatology Institute, Academic Health System, Hamad Medical Corporation, Doha, Qatar; ^3^Translational Research Institute, Academic Health System, Hamad Medical Corporation, Doha, Qatar; ^4^Weill Cornell Medicine-Qatar, Doha, Qatar; ^5^Qatar University, College of Medicine, Doha, Qatar; ^6^Department of Dermatology, Weill Cornell Medicine, New York, USA; ^7^College of Health and Life Sciences, Hamad Bin Khalifa University-Qatar

## Abstract

Atopic Dermatitis (AD) is a high-burden disease that affects approximately 2–5% of adults. AD patients experience intense pruritus and often report sleep and mental health disturbances accompanied by a diminished quality of life. The patients’ perceptions of their treatment benefits are becoming increasingly important in the benefit/risk assessment of therapeutics such as the gold standard in AD therapy, Dupilumab. A survey questionnaire (ADCT) has been recently developed to assess the control of AD symptoms using subjective patient-based reporting only. This study aimed to investigate the self-reported efficacy of Dupilumab in Qatari patients with severe AD using the new ADCT evaluation tool.

**Methods:** 30 patients completed a baseline survey before starting Dupilumab, and ADCT was assessed at four weeks post-therapy initiation. ADCT evaluates six AD symptoms in a severity grading from 0 to 3 (max. 24 points). The impact is assessed over the past week, including overall severity of symptoms, days with intense episodes of itching, the intensity of bother, problems with sleep, impact on daily activities, and impact on mood or emotions. In addition, itch severity was also assessed using a numeric rating scale (NRS11) ranging from 0 to 10.

**Results:** The overall mean ADCT score at baseline was 17.6, and at week 4, it was reduced to 4.1. Patients reported a dramatic change in the overall symptoms already in this early phase. The parried t-test showed a significant difference in ADCT Score before and after therapy. There was a substantial decline in experiencing the associated AD symptoms: overall severity of symptoms (mean baseline =3.1, Dupilumab week 4 =0.9 (3.1/0.9), days with intense episodes of itching (3.2/0.7), the intensity of bother (3.2/0.8), the problem with sleep (2.7/0.4), impact on daily activities (2.5/0.6), and impact on mood or emotions (2.9/0.6). The itch score also reduced from 8/10 at baseline to 0-3 at week 4.

**Conclusion:** Treatment of adult Asian/Arabic patients with severe AD treated with Dupilumab with or without topical steroids was highly effective and significantly improved overall well-being and pruritus as early as after 4 weeks of treatment.

